# Human Proteinase 3, an important autoantigen of c-ANCA associated vasculitis, shares cross-reactive epitopes with serine protease allergens from mites: an *in silico *analysis

**DOI:** 10.12688/f1000research.28225.2

**Published:** 2022-02-03

**Authors:** Emiro Buendía, Múnera Marlon, Orlando Parra, María Sánchez, Andrés Sánchez, Jorge Sánchez, Diego Viasus

**Affiliations:** 1 Department of Internal Medicine, Universidad del Norte, Barranquilla, Atlantico, 080004, Colombia; 2 Division of Health Sciences, Universidad del Norte, Barranquilla, Atlantico, 080004, Colombia; 3 Faculty of Medicine, Universidad de Cartagena, Cartagena, Colombia; 4 Medical Research group (GINUMED), Universitary Corporation Rafael Núñez, Cartagena, Bolívar, 130001, Colombia; 5 Department of Internal Medicine, Universidad El Bosque, Bogotá, Cundinamarca, 110111, Colombia; 6 Departement of Pediatrics, Universidad de Cartagena, Cartagena, Bolívar, 130001, Colombia; 7 Group of Clinical and Experimental Allergy (GACE), IPS Universitaria, Universidad de Antioquia, Medellín, Antioquia, 050001, Colombia

**Keywords:** Serine proteases, human proteinase 3, house dust mites group 3 allergens, ANCA associated vasculitis, sequence homology, T and B cell epitopes, cross-reactivity, epitope modelling

## Abstract

**Background: **In autoimmune vasculitis, autoantibodies to Human Proteinase 3 (PR3), a human serine protease, seems to have a role on the inception of c-ANCA associated vasculitis. The origin of this autoreactive response remains unclear. However, for several autoreactive responses, molecular mimicry between environmental antigens and human proteins is key to trigger autoantibodies and finally autoimmunity manifestations. Considering that PR3 is a serine protease and house dust mite (HDM) group 3 allergens share this biochemical activity, the aim of this study was to identify cross-reactive epitopes between serine proteases from human and mites using an *in silico* approach.

**Methods: **Multi alignment among amino acid sequences of PR3 and HDM group 3 allergens was performed to explore identity and structural homology. ElliPro and BepiPred *in silico* tools were used to predict B and T cell epitopes. Consurf tool was used to conduct identification of conserved regions in serine proteases family.

**Results: **PR3 and HDM group 3 allergens shared moderate identity and structural homology (root mean square deviation < 1). One B cell cross reactive epitope among serine proteases was identified (29I, 30V, 31G, 32G, 34E, 36K, 37A, 38L, 39A and 54C) and two T cell epitopes.

**Conclusions: **PR3 have structural homology and share cross reactive epitopes with HDM group 3 allergens.

## Introduction

Anti-neutrophil cytoplasmic antibody (ANCA)-associated vasculitis (AAV) is a life-threatening autoimmune disease affecting small vessels, compromising the respiratory mucosa, skin, lung, and the kidney ^1^. This group of small vessel vasculitis includes various diseases: granulomatosis with polyangiitis, microscopic polyangiitis, kidney-limited vasculitis and Eosinophilic granulomatosis with polyangiitis, all of them having in common some degree of autoimmune response to the Human Proteinase 3 protein (PR3). Previous studies have shown that autoantibody binding to PR3 expressed on the neutrophil surface may activate its degranulation, eliciting tissue damage in small vessels and their irrigated organs. Also, while proinflammatory effector T cells have been implicated in vasculitis pathogenesis ^2^, a specific PR3 T cell epitope has not been reported in AAV patients ^3^. PR3 is a serine protease physiologically expressed in human neutrophils. Due to its enzymatic activity, it degrades various intercellular gap-junction proteins and collagen and may play a role in neutrophil transendothelial migration. In addition, this protein is an important autoantigen in AAV, and sera from patients with severe and relapsing forms of the disease can bind it in IgG ELISA assays ^4– 6^. Further, although a cause-effect relationship between PR3-autoantibodies and vasculitis is not clearly defined, animal models support a pathogenic role ^7, 8^, revealing that they may be involved in disease inception, progression and severity ^1^.

Environmental exposures, specially to microbial components mimicking self-antigens have been proposed as triggers of autoimmunity ^9, 10^. Also, in AAV, it has been proposed that an endogenous immune response to a complementary protein to PR3 autoantigen could be implicated in disease inception, and this antisense protein harbors homology to various bacterial peptides ^11^. PR3 crystal structure has been elucidated, and various epitopes are recognized by patients suffering AAV; however, its cross-reactivity with environmental antigens is poorly studied ^12– 14^.

Previous studies have shown that specific IgE to some self-proteins have been identified in autoimmune and allergic diseases like lupus, urticaria, dermatitis, allergic pulmonary aspergillosis and have a strong association with disease activity ^15– 18^. Some allergens can cross-react with human proteins and participates in autoimmunity inception in pemphigus vulgaris by a “hit-and-run” mechanism, opening the theoretical possibility for a similar mechanism to occur in another autoimmune disease such as AAV ^19– 22^.

In the tropics, house dust mites (HDM) are important ubiquitous allergen sources and exposure is perennial, increasing the possibilities of exposure in the general population ^23^, and IgE sensitization to their components ^24, 25^. Sensitization to HDM group 3 allergens is common ^26^, as they harbor serine protease activity and conserved structural homology ^27^, making them potential PR3 cross reactive antigens; this has not been explored before. Here, we show *in silico* data suggesting cross-reactivity and epitope sharing between PR3 and HDM group 3 allergens.

## Methods

### Searching homologous with BLAST (Basic Local Alignment Search Tool)

The amino acid sequence from the human PR3 (Uniprot accession: P24158) was used as query to perform a search for serine protease homologous reported in allergenic sources: *Dermatophagoides pteronyssinus* (Der p 3: Accession number P39675), *Blomia tropicalis* (Blo t 3: A1KXI1), *Glycyphagus domesticus* (Gly d 3: Q1M2M8), *Lepidoglyphus destructor* (Led p 3: Q1M2L7) and *Tyrophagus putrescentiae* (Tyr p 3: C6ZDB5) with the PSI-BLAST tool. Parameters were set as default.

### Multiple alignment

Identity among all allergenic sequences homologous to PR3 was analyzed using the Jalview tool2.11.0 ^28^. First, all allergens and human PR3 codes were used as inputs in the Jalview tool. Second, the T coffee tool was chosen to assess alignment. Third, alignment was displayed as an identity percentage.

### Construction of 3D model

The 3D model of Der p 3, a serine protease of *Dermatophagoides pteronyssinus* was generated by homology in the SWISS-MODEL server using the zymogen catalytic region of human MASP-2 (PDB: 1zjk f) as a template. The 3D model of Der p 3 was loaded into the ProSA-web server ^29^, which was used to analyze its quality.

The model was refined in DeepView v4.1 (energy minimization and rotamer replacements). Its quality was evaluated by several tools, including Ramachandran graphs, WHATIF, QMEAN4 index, and energy values (GROMOS96 force field). For the validation of the Der p 3 structure we used the Minimize Structure option in the UCSF Chimera software, a procedure that adjust the energy and reduce the entropy of the model ^30^.

Three-dimensional structure (PDB: 1FUJ) of the human PR3 serine protease was retrieved from the Protein Data Bank. A cartoon model was created using Pymol software v2.4. Root median square deviation (RMSD) value between Der p 3 and PR3 was calculated using Chimera software v1.0 ^30^.

### B and T cell epitope prediction

ElliPro v3.0 and BepiPred v2.0 tools were used to predict B and T cell epitopes on Der p 3 ^31, 32^. With ElliPro, the 3D structure of Der p 3 was used to predict epitopes. Minimum score and maximum distance (Angstrom) were set to 0.5 and 6, respectively. Epitopes with high conserved rates were visualized in the 3D model. For prediction using BepiPred, an amino acid sequence of Der p 3 was used as input.

### Conservation analysis

The 3D structure of Der p 3 was submitted to the ConSurf server to generate evolutionarily related conservation scores to help to identify functional regions in the proteins. HMMER algorithm, 1 iteration, E-value cutoff (0.0001) and UNIREF-90 database was set as default to generate multiple alignment, prior to evolutive analysis. All amino acid sequences in FASTA format were used.

## Results

### Human PR3 and HDM group 3 allergens exhibited identity and features of the serine protease family

**Figure 1.**
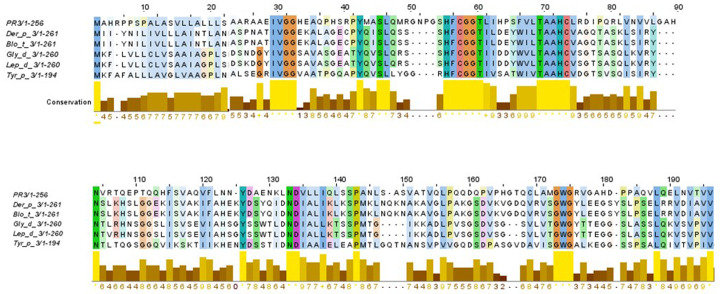
Multiple alignment among PR3 and the HDM allergens belonging to group 3. An identity of 45% in their amino acid sequences was found.

BLAST search identified various serine protease family members from HDM as homologous. The multiple sequence alignment analysis showed that Der p 3, Blo t 3, Gly d 3, Led p 3 and Tyr p 3 allergens shared 45% of identity in their aminoacid sequences with PR3. The most conserved region is located between residues 53 to 75, indicating the existence of molecular mimicry ( Figure 1). Among the members of HDM group 3 allergens, an identity until 41% was reported ( Table 1), and a highly conserved region between residues 40 to 90 was found. When identity between PR3 and each allergen used in study was analyzed, a moderate level of identity was found (30%) ( Table 1).

**Table 1.** Identity matrix among serine proteases used in study. All comparisons of PR3 with HDM group 3 allergens showed a moderate identity.

	**PR3**	**Der p 3**	**Blo t 3**	**Gly d 3**	**Lep d 3**	**Tyr p 3**
**PR3**	100	33	27	30	30	27
**Der p 3**	33	100	48	52	53	43
**Blo t 3**	27	48	100	58	58	47
**Gly d 3**	30	52	58	100	99	41
**Lep d 3**	30	53	58	99	100	41
**Tyr p 3**	27	43	47	41	41	100

**Figure 2.**
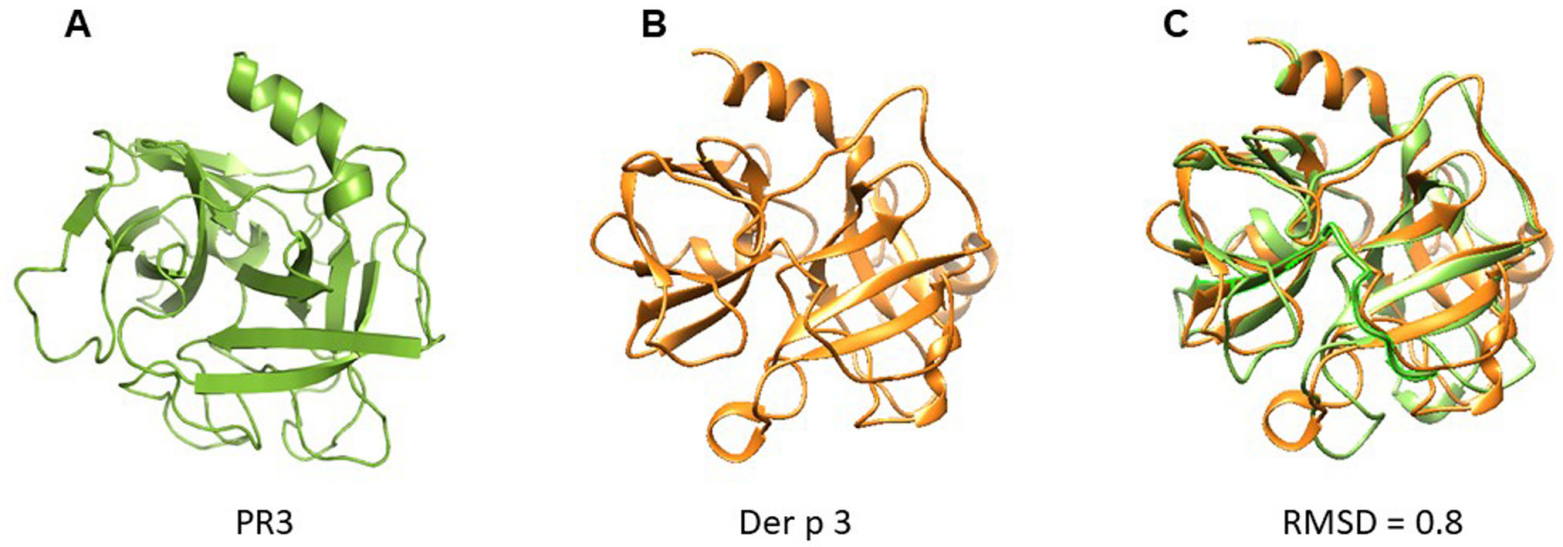
Cartoon models of PR3 and Der p 3. ( **A**) PR3; ( **B**) Der p 3. According to modelling by homology, Der p 3 exhibited a typical fold of serine protease family. ( **C**) Overlapping of 3D model of PR3 and Der p 3. RMSD value of 0.8 is reported, suggesting structural homology.

A structural model of Der p 3 was obtained by homology modelling using the 3D structure of PR3 reported in the PDB database. According to modelling, the Der p 3 tertiary structure exhibited a typical fold of serine protease family, conformed by four α-helixes and fifteen β-strands with structural homology with PR3 (RMSD = 0.8) ( Figure 2). 

### T and B cell cross-reactive epitopes were predicted between HDM group 3 allergens and PR3

**Figure 3.**
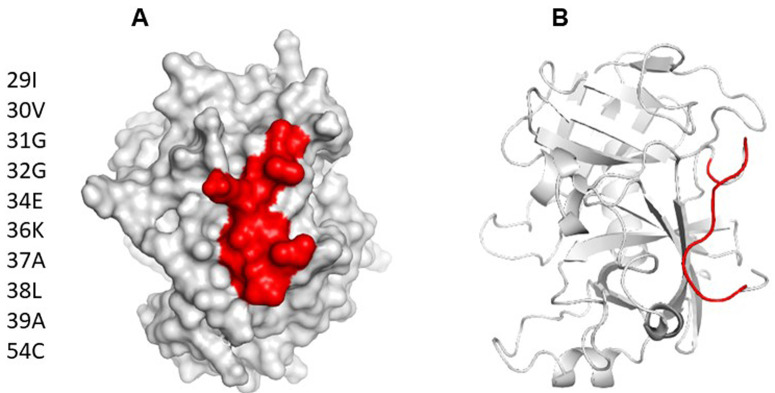
( **A**) Surface model of Der p 3, showing area occupied by B cell epitope predicted as cross reactive. ( **B**) Cartoon model showing location of epitope on tridimensional structure. It can be appreciated that the predicted epitope is on a loop spanning residues 29 to 39.

**Figure 4.**
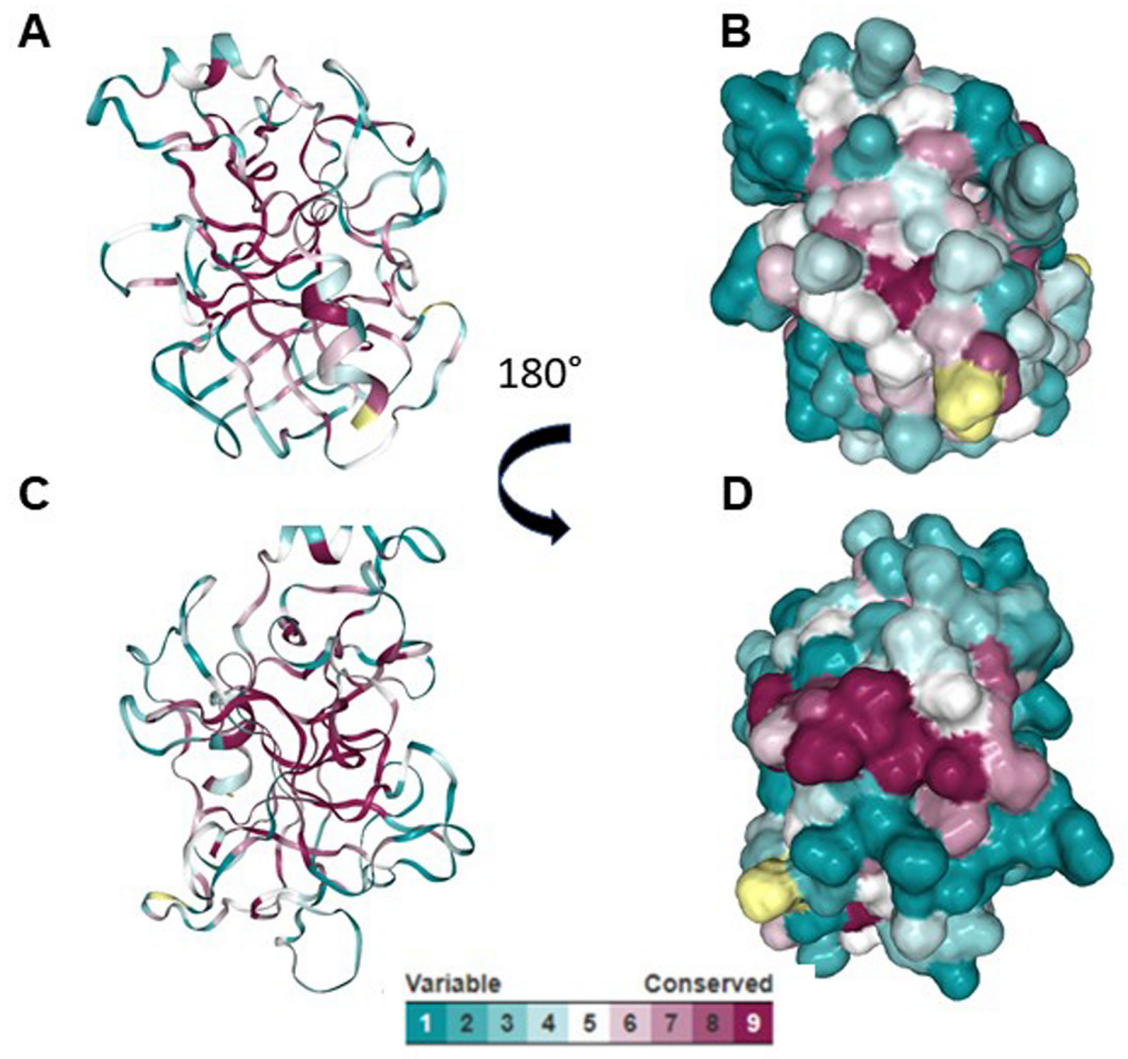
Phylogenetic analysis of the serine proteases using Consurf. ( **A** and **C**) Cartoon models showing the conserved region among serine proteases. ( **B** and **D**) Surface models showing the conserved region among serine proteases.

**Figure 5.**
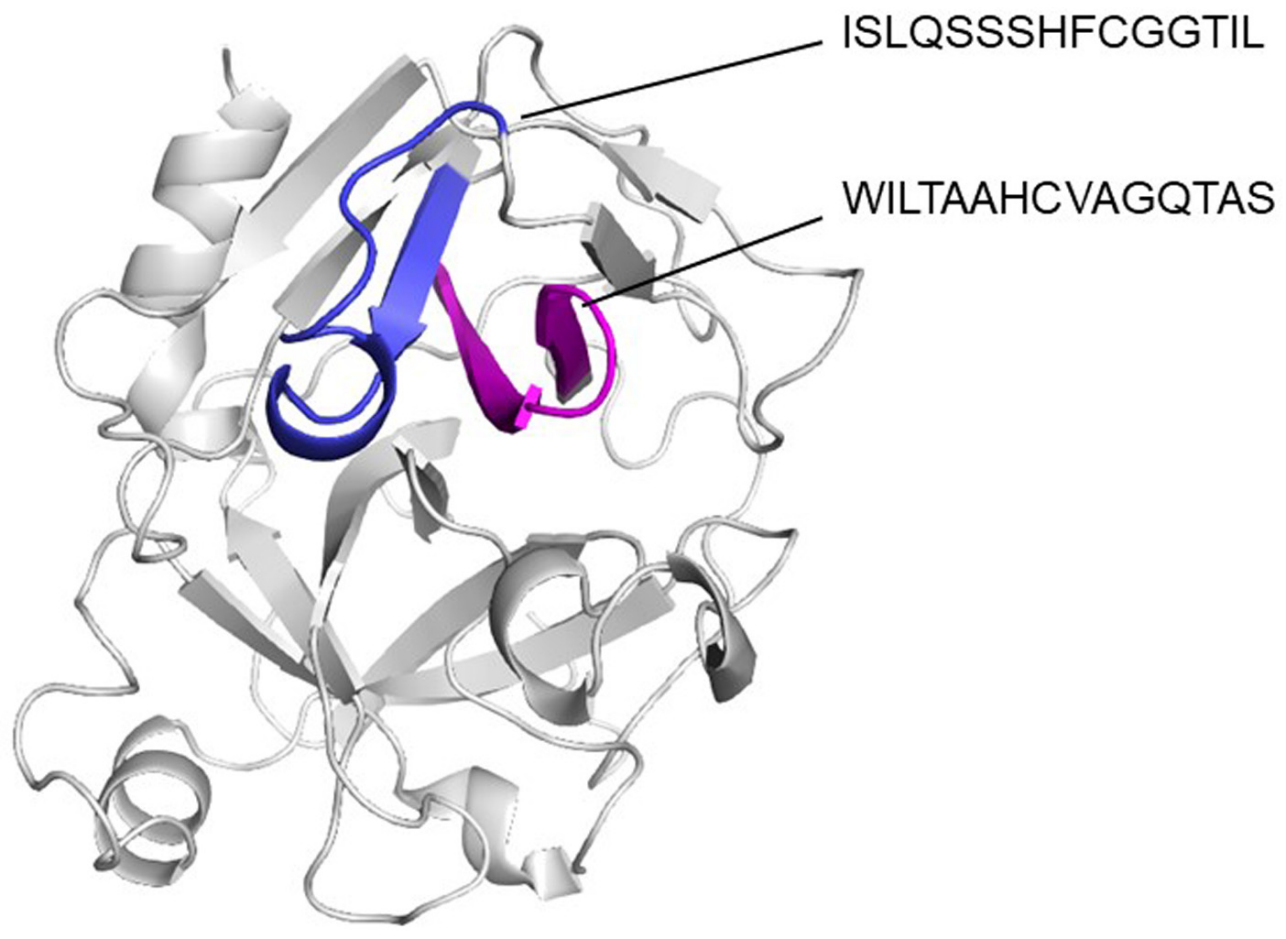
Cartoon model showing the location of T cell epitopes in their tridimensional structure. It can be appreciated that predicted epitopes are in a continuous β strands (blue and magenta).

Using ElliPro and BepiPred servers, a cross reactive B cell epitope was predicted on all serine protease used in this study. This epitope is formed by ten residues and is on the N-terminal region, spanning amino acids 29 and 39 with a surface area of 470 Å, not forming part of any domain within the protein. Conservative analysis indicated that the antigenic region predicted was highly conserved in the serine proteases ( Figure 3). According to ConSurf analysis, the region covering the cross-reactive epitope is conserved among the serine protease family ( Figure 4). T cell epitope prediction identified at least two epitopes with potential cross-reactivity among all sequences analyzed. Both epitopes are located on the first and second β strands: the first epitope spans the 45 to 59 region (ISLQSSSHFCGGTIL); and the second, the 63 to 77 region (WILTAAHCVAGQTAS) ( Figure 5; Table 2).

**Table 2.** T cell epitopes predicted *in silico*. Both are conserved among PR3 and HDM group 3 allergens.

Allele	Start	End	Sequence
HLA-DRB1*01:01	45	59	ISLQSSSHFCGGTIL
HLA-DRB1*01:01	63	77	WILTAAHCVAGQTAS

## Discussion

In this study we found that PR3 and HDM group 3 serine protease allergens have conserved identity and homology. Also, for the first time, we predicted various T and B cell cross reactive epitopes between them through an *in silico* approach. PR3 is an important autoantigen in small vessel vasculitis and it seems to participate in disease inception, progression, and severity ^1^. Our results have potential implications for the understanding of autoreactive response in AAV and open the possibility for a new environmental trigger of the autoreactive response in AAV.

In AAV, it has been proposed that autoantibodies directed to a complementary protein to PR3 autoantigen could be implicated in disease inception, and this antisense protein harbors homology to various bacterial peptides ^11^ – a theory named autoantigen complementarity ^33^. However, in epidemiological studies, autoantigen complementarity hypothesis testing has showed conflicting results, since sera from some patients suffering from AAV do not recognize complementary PR3, while others do ^34– 36^. Also, molecular mimicry of PR3 protein by infectious microorganism components have been proposed as a possible environmental trigger of the disease based on the reports of infections preceding the manifestations of vasculitis ^10, 37– 39^, although a cross reactive antigen have not been reported yet.

In their seminal publication, Pendergraft *et al*. run a BLAST query to find homologues of PR3 protein in microbial or fungal microorganisms, and do not find matching sequences at that time ^11^. However, they do not include Arachnida or other environmental sources of cross-reactivity. In our analysis we find matching PR3 protein sequences with various HDM group 3 serine protease allergens, and at least theoretically this finding could have many implications for the understanding of inception and even diagnosis of autoreactive response in AAV. Recently, Qian *et al*. have shown that some allergens can cross-react with human proteins ^19^ and participate in autoimmunity inception in pemphigus vulgaris by a “hit-and-run” mechanism ^22^, opening the theoretical possibility for a similar mechanism to occur in another autoimmune disease such as AAV. Similarly, in atopic dermatitis, Valenta and collaborators observe that some patients with severe complications from the disease, had IgE directed to the profilin of the *Betula verrucosa*, but also to the human homologue ^40^.

In the tropics, HDM are important ubiquitous sources of protease allergens. Exposure is perennial, increasing the possibilities of exposure and IgE sensitization to their components in the general population ^23– 25^. Sensitization to HDM group 3 allergens is common ^26^, and they harbor serine protease activity ^27^, a characteristic that make them highly allergenic. Moreover, their conserved structural homology makes them highly immunogenic ^41, 42^ and suitable for epitope spreading ^43^. In this context, “hit-and-run” and epitope spreading establish framework mechanisms for environmental allergens with homology to autoantigens to potentially participate in the development of autoimmunity. We speculate that HDM group 3 allergens harbor two characteristics that make them suitable candidates for environmental triggering of AAV: their proteolytic activity that, as other protease allergens, set a tissue damaging microenvironment during antigen recognition ^41^; and molecular homology-epitope sharing with human PR3, that would elicit B cell autoantibody production and autoreactive T cell receptor generation. In conclusion, we observe that PR3 and HDM group 3 serine protease allergens have conserved identity, and for the first time we predict cross-reactive epitopes between them through an *in silico* approach.

## Data availability

UniProtKB: PRTN3_HUMAN, Accession number P24158: https://www.uniprot.org/uniprot/P24158

Protein Data Bank: PR3 (MYELOBLASTIN), Accession number 1FUJ: https://www.rcsb.org/structure/1FUJ

UniProtKB: Mite allergen Der p 3, Accession number P39675: https://www.uniprot.org/uniprot/P39675

UniProtKB: Trypsin Blo t 3, Accession number A1KXI1: https://www.uniprot.org/uniprot/A1KXI1

UniProtKB: Gly d 3, Accession number Q1M2M8: https://www.uniprot.org/uniprot/Q1M2M8

UniProtKB: Allergen Lep d 3, Accession number Q1M2L7: https://www.uniprot.org/uniprot/Q1M2L7

UniProtKB: Trypsin Tyr p 3.0101, Accession number C6ZDB5: https://www.uniprot.org/uniprot/C6ZDB5
