# Supplementary material for: Human Proteinase 3, an important autoantigen of c-ANCA associated vasculitis, shares cross-reactive epitopes with serine protease allergens from mites: an in silico analysis
Source: F1000Res. 2022 Feb 3;10:47. Originally published 2021 Jan 26. [Version 2] doi: 10.12688/f1000research.28225.2 (PMC9099154; doi:10.12688/f1000research.28225.2)
Supplement: Peer review history

# Peer review: Reviewer response for version 2

**DOI:** 10.5256/f1000research.81402.r136303

**Published:** 2022-05-12

**Authors:** Hiroshi Tanaka

**Affiliations:** 1 Department of School Health Science, Hirosaki University Hospital, Hirosaki, Japan

To elucidate the origin of the autoreactive response of human proteinase 3 (PR3), the authors examined molecular similarity between environmental antigens and PR3. They found that some house dust mites showed structural homology to PR3. Some environmental antigens, such as house dust mites, may be attributable to the production of PR3-ANCA.

 This is an interesting paper. The results obtained is informative enough for readers.

 It is nice to add some comments or speculation about how the results obtained are relevant in real-world clinical practice, particularly the etiology and treatment of patients with C-ANCA-related vasculitis. This issue is very relevant for physicians who treat patients with ANCA-related vasculitis.

Is the work clearly and accurately presented and does it cite the current literature?

Yes

If applicable, is the statistical analysis and its interpretation appropriate?

Not applicable

Are all the source data underlying the results available to ensure full reproducibility?

Yes

Is the study design appropriate and is the work technically sound?

Yes

Are the conclusions drawn adequately supported by the results?

Yes

Are sufficient details of methods and analysis provided to allow replication by others?

Yes

Reviewer Expertise:

Nephrology, Rheumatology, Clinical immunology, Pediatrics

I confirm that I have read this submission and believe that I have an appropriate level of expertise to confirm that it is of an acceptable scientific standard.

# Peer review: Reviewer response for version 2

**DOI:** 10.5256/f1000research.81402.r124319

**Published:** 2022-03-29

**Authors:** Tomas Erban

**Affiliations:** 1 Crop Research Institute, Prague, Czech Republic

Buendia et al. investigated in their manuscript possible link between human proteinase 3 (PR3) and house dust mite allergen protease that is denoted as allergen group 3. Authors suggest that similarity between PR3 and the group 3 mite allergens may be link to autoimmune vasculitis. Methodology and conclusions are based on structural homology based on in silico analysis. I believe that recommendations will improve the manuscript, which is of interest.

 Because methodology of the study is based on in silico tools, it is necessary to consider that the function is “possible” and needs to be confirmed in future. 

 Please note, that group 3 mite allergens become subject of debate in the domestic mites, that include both hose dust (HDMs) and stored product mite (SPMs) species. Immortally, authors should clarify that allergens of HDMs and SPMs were included in the study. In addition, these allergens are present in mite feces and, therefore, they are abundant in the environment. Thus, introduction should be improved.

 It was found that occurrence of trypsin allergens in mites differs in species and strains. The expression of group 3 mite allergens was observed to be affected by presence of intracellular symbionts in mites. There is suggested link to PAR2 (PAR-2) in mites (– for instance, see: https://doi.org/10.1016/j.jprot.2021.104356 ^1^). The affected pathway in mites can be affected also in humans. Could be a suggested link to PR3 in this regard? e.g. “immune functions of PR3 with respect to PR3 modulation of cell activation via cleavage of protease-activated receptor-2 (PAR-2)” - Ann N Y Acad Sci. 2007 Aug;1109:84-92. doi: 10.1196/annals.1398.010 ^2^.

 Authors can also consider the suggested “Orchestration of an uncommon maturation cascade of the house dust mite protease allergen quartet” (https://doi.org/10.3389/fimmu.2014.00138 ^3^)

 Comment to approach 

- Authors used different sequences of group 3 mite allergens to predict similarity. It should be clear whether “pro” or mature form was used in analyses. It is recommended that authors will consider change “identity” to similarity. Usually, high identity is “only” to partial sequence. It is important to consider overall similarity.
- In initial stage, authors used multiple Grp3 sequences, i.e.: Der p 3, Blo t 3, Gly d 3, Led p 3 and Tyr p 3. Is it possible to report similarity? 
- In further analysis, authors used Der p 3 sequence. Incidentally, research showed that D. pteronyssinus is depleted of this allergen in mite feces, although it is high-abundance in D. farinae – for instance, see J Proteomics. 2017 Jun 6;162:11-19  doi: 10.1016/j.jprot.2017.04.021 ^4^

Is the work clearly and accurately presented and does it cite the current literature?

Partly

If applicable, is the statistical analysis and its interpretation appropriate?

Partly

Are all the source data underlying the results available to ensure full reproducibility?

Yes

Is the study design appropriate and is the work technically sound?

Partly

Are the conclusions drawn adequately supported by the results?

Partly

Are sufficient details of methods and analysis provided to allow replication by others?

Yes

Reviewer Expertise:

Invertebrate physiology, proteomics, acarology, allergens, multiomics

I confirm that I have read this submission and believe that I have an appropriate level of expertise to confirm that it is of an acceptable scientific standard, however I have significant reservations, as outlined above.

## References

[1] : Label-free proteomic analysis reveals differentially expressed Wolbachia proteins in Tyrophagus putrescentiae: Mite allergens and markers reflecting population-related proteome differences. *Journal of Proteomics* .2021;249: 10.1016/j.jprot.2021.104356 10.1016/j.jprot.2021.104356 34438106

[2] : Human proteinase 3 (PR3) and its binding molecules: implications for inflammatory and PR3-related autoimmune responses. *Ann N Y Acad Sci* .2007;1109: 10.1196/annals.1398.010 84-92 10.1196/annals.1398.010 17785293

[3] : Orchestration of an uncommon maturation cascade of the house dust mite protease allergen quartet. *Front Immunol* .2014;5: 10.3389/fimmu.2014.00138 138 10.3389/fimmu.2014.00138 24744761PMC3978338

[4] : Detailed two-dimensional gel proteomic mapping of the feces of the house dust mite Dermatophagoides pteronyssinus and comparison with D. farinae : Reduced trypsin protease content in D. pteronyssinus and different isoforms. *Journal of Proteomics* .2017;162: 10.1016/j.jprot.2017.04.021 11-19 10.1016/j.jprot.2017.04.021 28442447

# Peer review: Reviewer response for version 1

**DOI:** 10.5256/f1000research.31220.r90330

**Published:** 2021-08-09

**Authors:** Chandrabose Selvaraj

**Affiliations:** 1 Department of Bioinformatics, Computer Aided Drug Design and Molecular Modeling Lab, Alagappa University, Karaikudi, Tamilnadu, 630003, India

Reviewer Recommendation: Major Revision

1. Primary check on this manuscript for language refinement is mandatory as I see that it is not satisfactory, and moreover, there are more typographical errors. I suggest the authors go with language and grammar checks.
2. The following sentence seems incomplete and difficult to understand, reframe it “Here, allergens such as: Der p 3, Blo t 3, Gly d 3, Led p 3 and Tyr p 3 shared an identity of 45% among them according to multiple sequence alignment"
3. Why have authors selected PR-3 for the homologous evaluation study? Since the 3-D structure is available in the PDB database?
4. What basis authors have selected allergenic sources such as Dermatophagoides pteronyssinus (Der p 3: Accession number P39675), Blomia tropicalis (Blo t 3: A1KXI1), Glycyphagus domesticus (Gly d 3: Q1M2M8), Lepidoglyphus destructor (Led p 3: Q1M2L7) and Tyrophagus putrescentiae (Tyr p 3:C6ZDB5) in this study? In BLAST it did not show the above mentioned allergens. 
5. Did authors find the sequence similarity analysis of target sequence Der p 3 with its template; if so provide that information more in detail.
6. Swiss-Modeler does not show the 1FUJ as template in template searching, and then what basis authors have checked RMSD calculation between the modeled structure and IFUJ as template?
7. In methodology part authors have mentioned that the modeled structure was subjected to validation, but lack results and interpretation. Provide more in detail.
8. Provide abbreviation for AAV
9. Is the residues present in the epitope region responsible for the antigenic effect?
10. In epitope prediction, the author only provides the sequence and length of the epitopes, if the author provides the information on score value, identity and whether it is present in any domain or motif it could be more informative. 

Is the work clearly and accurately presented and does it cite the current literature?

Yes

If applicable, is the statistical analysis and its interpretation appropriate?

No

Are all the source data underlying the results available to ensure full reproducibility?

Partly

Is the study design appropriate and is the work technically sound?

Partly

Are the conclusions drawn adequately supported by the results?

Partly

Are sufficient details of methods and analysis provided to allow replication by others?

No

Reviewer Expertise:

Immunoinformatics, Computational Biology, Molecular Modeling

I confirm that I have read this submission and believe that I have an appropriate level of expertise to confirm that it is of an acceptable scientific standard, however I have significant reservations, as outlined above.

# Response

**Published:** 2021-12-10

**Authors:** Emiro Buendía

**Affiliations:** Universidad del Norte, Colombia, Colombia

**Emiro Buendía, **Division of Health Sciences, Universidad del Norte, Barranquilla, Colombia.

 Dear reviewer, thanks for the reading of the paper, we have taken note of all your comments, questions and suggestions. Our responses are bellow:

**1. Reviewer:** Primary check on this manuscript for language refinement is mandatory as I see that it is not satisfactory, and moreover, there are more typographical errors. I suggest the authors go with language and grammar checks.

**Answer:** We appreciate your suggestion. The manuscript was revised and refined by a language style corrector.

**2. Reviewer:** The following sentence seems incomplete and difficult to understand, reframe it “Here, allergens such as: Der p 3, Blo t 3, Gly d 3, Led p 3 and Tyr p 3 shared an identity of 45% among them according to multiple sequence alignment"

**Answer:** We appreciate your suggestion. A new sentence was added: “Multiple sequence alignment showed that Der p 3, Blo t 3, Gly d 3, Led p 3 and Tyr p 3 allergens shared 45% of identity in their aminoacid sequences with PR3. The most conserved region is located between residues 53 to 75, indicating the existence of molecular mimicry.”

**3. Reviewer:** Why have authors selected PR-3 for the homologous evaluation study? Since the 3-D structure is available in the PDB database?

**Answer:** We appreciate your question. We selected PR3 for the study because it is the most important autoantigen in ANCA + vasculitis. Since, this human protein is a serine protease, in the sequence alignment analysis we seek for other serine proteases in mites, because it is a previously described environmentally ubiquitous serine protease source. In our study we selected the 3D model of PR3 from the Protein Data Bank as mentioned in your observation because its structure was already deciphered, the structural homology analysis was done keeping in mind that its serine protease folding could make more possible for us to model homologous serine in mites.

**4. Reviewer:** What basis authors have selected allergenic sources such as Dermatophagoides pteronyssinus (Der p 3: Accession number P39675), Blomia tropicalis (Blo t 3: A1KXI1), Glycyphagus domesticus (Gly d 3: Q1M2M8), Lepidoglyphus destructor (Led p 3: Q1M2L7) and Tyrophagus putrescentiae (Tyr p 3:C6ZDB5) in this study? In BLAST it did not show the above-mentioned allergens.

**Answer:** We appreciate your question. These allergens were chosen on the basis that they belong to the serine protease family, and our hypothesis was that the sensitization to these allergens is cross-reactive with the PR3 autoantigen. So, search was limited using terms: American house dust mite (taxid:6954). In this way, results were only about mites, an important allergenic source.

**5. Reviewer:** Did authors find the sequence similarity analysis of target sequence Der p 3 with its template; if so provide that information more in detail.

**Answer:** We appreciate your question. Yes, the binary alignment showed 33% identity between the template amino acid sequences, and Der p 3. An image of the alignment was uploaded in the new manuscript version.

**6. Reviewer:** Swiss-Modeler does not show the 1FUJ as template in template searching, and then what basis authors have checked RMSD calculation between the modeled structure and IFUJ as template?

**Answer:** We appreciate your question. For modelling the mite allergen Der p 3 structure, we used the zymogen catalytic region of MASP-2, a lectin binding mannose from human (PDB: 1zjk f), as a template because 1FUJ code was not listed in Swiss-Modeler query results for this protein. Next we calculated the RMSD based on the predicted structure of the mite Der p3 protease and the previously resolved structure of  the human PR3. The following sentence was improved to answer your question in the method section of the paper ( **Construction of 3D model**): “The 3D model of Der p 3, a serine protease of *Dermatophagoides pteronyssinus* was generated by homology in the SWISS-MODEL server using the zymogen catalytic region of human MASP-2 (PDB: 1zjk f) as a template. The 3D model of Der p 3 was loaded into the ProSA-web server ^29^ , which was used to analyze its quality”.

**7. Reviewer:** In methodology part authors have mentioned that the modeled structure was subjected to validation, but lack results and interpretation. Provide more in detail.

**Answer:** We appreciate your suggestion. We added the next sentence in the method section ( **Construction of 3D model**): For the validation of the Der p 3 structure we used the Minimize Structure option in the UCSF Chimera software, a procedure that adjust the energy (entropy) of the model. 

**8. Reviewer:** Provide abbreviation for AAV.

**Answer:** We appreciate your suggestion. ANCA Associated Vasculitis (AAV) was included in the corrected paper text.

**9. Reviewer:** Is the residues present in the epitope region responsible for the antigenic effect?

**Answer:** We appreciate your question. We think that the residues found in the epitope region are involved in the cross reactivity between the studied serine protease and hypothesized have a role in autoimmunity inception. But this are preliminary results awaiting experimental validation. 

**10. Reviewer:** In epitope prediction, the author only provides the sequence and length of the epitopes, if the author provides the information on score value, identity and whether it is present in any domain or motif it could be more informative. 

**Answer:** We appreciate your suggestion. The B epitope is part of the N-terminal region of PR3 and is not within any domain of the protein. The T epitope spanned between residues 45-59 is located on the first and second beta lamina. While the second epitope T (63-77) is located in the third beta sheet, and first alpha helix in the protein structure. In the result section ( **T and B cell cross-reactive epitopes were predicted between HDM group 3 allergens and PR3** **), **two sentences were improved to consider your suggestion.
